# Lipid Droplet-Organelle Contact Sites as Hubs for Fatty Acid Metabolism, Trafficking, and Metabolic Channeling

**DOI:** 10.3389/fcell.2021.726261

**Published:** 2021-09-14

**Authors:** Mike F. Renne, Hanaa Hariri

**Affiliations:** ^1^Sir William Dunn School of Pathology, University of Oxford, Oxford, United Kingdom; ^2^Department of Biological Sciences, Wayne State University, Detroit, MI, United States

**Keywords:** fatty acids, lipid droplets, contact sites, metabolism, organelles

## Abstract

Cells prepare for fluctuations in nutrient availability by storing energy in the form of neutral lipids in organelles called Lipid Droplets (LDs). Upon starvation, fatty acids (FAs) released from LDs are trafficked to different cellular compartments to be utilized for membrane biogenesis or as a source of energy. Despite the biochemical pathways being known in detail, the spatio-temporal regulation of FA synthesis, storage, release, and breakdown is not completely understood. Recent studies suggest that FA trafficking and metabolism are facilitated by inter-organelle contact sites that form between LDs and other cellular compartments such as the Endoplasmic Reticulum (ER), mitochondria, peroxisomes, and lysosomes. LD-LD contact sites are also sites where FAs are transferred in a directional manner to support LD growth and expansion. As the storage site of neutral lipids, LDs play a central role in FA homeostasis. In this mini review, we highlight the role of LD contact sites with other organelles in FA trafficking, channeling, and metabolism and discuss the implications for these pathways on cellular lipid and energy homeostasis.

## Introduction

Maintaining energy homeostasis is obligatory for cellular survival and fitness. In general, cells store energy in the form of fatty acids (FAs) when nutrients are abundant or in excess. Stored FAs can be released under stress conditions or when nutrients are limited, in order to be used for the synthesis of membrane lipids or to fuel energy production. FAs are stored in the form of neutral lipids, with triacylglycerol (TAG) and sterol esters (SE) being the most abundant in most cells. These neutral lipids are incapable of forming membranes; therefore, they are sequestered in specific organelles known as Lipid Droplets (LDs) that form at the ER (Fujimoto and Ohsaki, 2006; Walther et al., 2017; Olzmann and Carvalho, 2019). LDs have a unique structure, consisting of a hydrophobic neutral lipid core, coated by a lipid monolayer studded with proteins (Tauchi-Sato et al., 2002). Different enzymes and proteins are targeted to the surface of LDs which reflects their functional heterogeneity (Kory et al., 2016; Bersuker et al., 2018; Prévost et al., 2018).

LDs are dynamic organelles that are regulated in response to cellular and physiological conditions. As the storage site of neutral lipids, LDs play a central role in energy and lipid metabolism. LD size, number, and composition vary depending on the cell type, nutrient availability, and metabolic state (Thiam and Beller, 2017). LD growth has been linked to human diseases including obesity and hepatic steatosis (Dietrich and Hellerbrand, 2014; Xu et al., 2016; Sans et al., 2019). In general, many cell types increase LDs number and size when a carbon source (glucose or fats) is abundant, as a results of increased NL production. *Vice versa*, under carbon restriction, stored FAs are utilized, leading to reduction in LD size and number. Notably, it was also reported that during prolonged periods of nutrient deprivation when autophagy is activated, the amount of LDs increases sequestering free FAs released during autophagic degradation of membranous organelles. This provide a buffering system to reduce lipotoxic accumulation of free FAs (Nguyen et al., 2017). Furthermore, there are notable examples where LD size and number do not correlate. For instance, in non-alcoholic fatty liver disease hepatocytes can present with either an increased numbers of large LDs (marcovesicular steatosis) or very small LDs (microvesicular steatosis) (Tandra et al., 2011; Takahashi and Fukusato, 2014). Finally, LD size and number are influenced by cellular processes that are not necessarily related to cellular nutrient status, such as LD fusion. These events are mediated by specialized proteins that enrich at the interface where LDs interact with each other (Gong et al., 2011; Xu et al., 2016; Sans et al., 2019). For example, in adipocytes, Fsp27 promotes directional transfer of TAG from smaller to larger LDs to promote LD growth (Gong et al., 2011).

**FIGURE 1:**
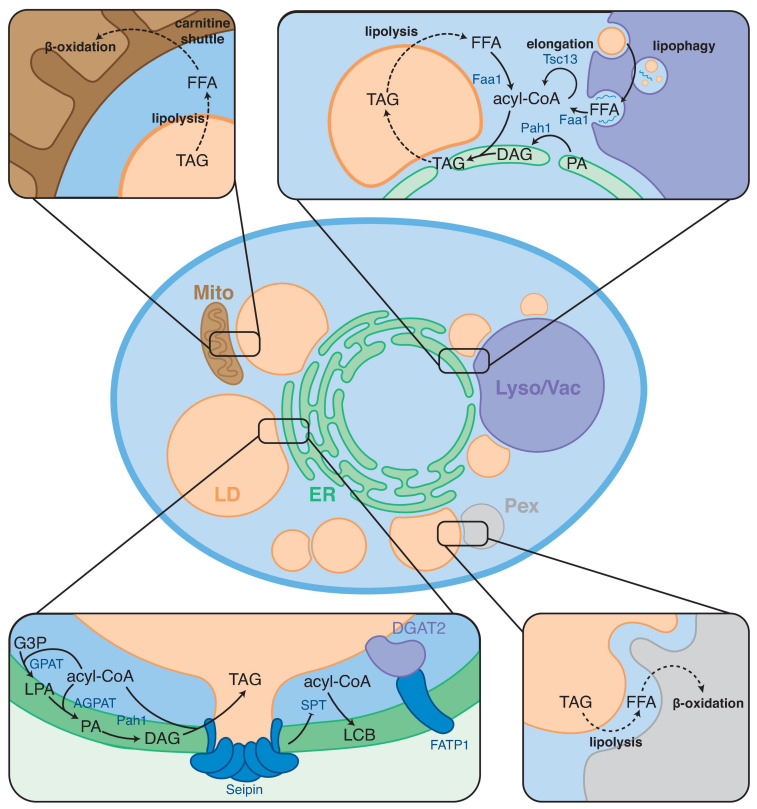
Overview of FA metabolic steps taking place at LD contact sites. LDs form contact sites with the endoplasmic reticulum (ER), mitochondria (Mito), peroxisomes (Pex), and lysosome/vacuole (Lyso/Vac). Boxes show examples of FA metabolic processes taking place at these contact sites. Arrows indicate enzymatic conversions; dashed arrows indicate trafficking/transport steps.

LDs as neutral lipid storage site are instrumental in FA metabolism, and LD contact sites are thought to play a key role in coordinating FA synthesis, storage, release, and breakdown. The role of LDs in FA metabolism is highlighted under stress conditions. For example, when cells accumulate excess FAs, efficient regulation of FAs flux to LDs is required to prevent lipotoxicity (Listenberger et al., 2003; Petschnigg et al., 2009). As many processes involved in energy and lipid metabolism are not localized to LDs, close collaboration and communication with other organelles is required. To this extent, LDs form contact sites with various organelles (Gao and Goodman, 2015; Schuldiner and Bohnert, 2017; Figure 1). Primarily, LDs have contacts with the ER, which supplies the bulk of the LD constituents. In addition, LDs form contact sites with mitochondria, peroxisomes, other LDs, and lysosomes (mammals) or the vacuole (yeast) (Valm et al., 2017; Shai et al., 2018). These contacts play important roles in maintaining energy homeostasis and balancing LD synthesis and turnover to maintain the complex cellular needs. Additionally, LD-organelle contacts form metabolic hubs that regulate LD biogenesis, growth, and distribution. Interestingly, the cellular localization of LDs and the sites of LD biogenesis have recently been indicated to respond to the cellular metabolic state (Hariri et al., 2018; Ugrankar et al., 2019; Henne et al., 2020). Consequently, proteins involved in maintaining LD contact sites have been implicated in various metabolic disorders (Herker et al., 2021).

FA metabolism requires extensive collaboration between different intracellular organelles. Although many of the enzymes and transporters involved in FA metabolism have been identified, major questions remain regarding how FA metabolic pathways are spatially organized and coordinated such that they can respond to the diverse, ever-changing physiological demands of cells. It has been previously proposed that the efficiency of metabolic pathways can be enhanced by organizing sequential enzymes of a metabolic process into transient functional complexes called metabolons (Srere, 1987; Ovádi and Sreret, 1999). Enzymes in a metabolon cooperate and efficiently hand-over substrates and products without releasing them to the bulk cytosol, thereby increasing local concentrations and stimulating metabolic reactions. This process is known as “metabolic channeling.” To date, several proteins have been reported to reside and function at LD-organelle contact sites possibly functioning in the formation of neutral lipid metabolon to locally regulate FA metabolism (Henne et al., 2020). These proteins include lipid-transfer proteins and FA-modifying enzymes suggesting that LD-organelle sites are specialized cellular locales where FA metabolism is compartmentalized. This mini review focuses on key FA metabolic processes that occur specifically at LD-organelle contact sites. First, we briefly overview the biochemical pathways involved in bulk FA synthesis and metabolism. Then, we describe the current knowledge of how these pathways and their enzymes are spatially organized at LD-organelle contact sites. Lastly, we discuss the implications of this organization on cellular metabolism and lipid homeostasis.

## Fatty Acid Metabolism at a Glance

### Fatty Acid Synthesis, Desaturation, and Elongation

Bulk FAs are synthesized *de novo* by the fatty acid synthase (FAS) complex as acyl-CoA, and this process takes place in the cytosol. FA synthesis starts from acetyl-CoA (C_2_-CoA), which is elongated in a cyclic reaction using malonyl-CoA (a C_3_-CoA) as the carbon chain donor. Malonyl-CoA is synthesized from acetyl-CoA by the acetyl-CoA carboxylase (ACC) enzymes, and production of this substrate is a rate-limiting step for FA synthesis.

FA synthesis starts with ligation of the acetyl- and malonyl- to an acyl carrier protein (ACP), by a trans-acylase enzyme. Acetyl-ACP is elongated with the addition of two carbon atoms in a cyclic cascade of four subsequent reactions. First, the (C_*n*_) acyl chain is coupled to the malonyl-ACP in a condensation reaction, yielding 3-ketoacyl-ACP. The 3-keto group is reduced to an alcohol, yielding 3-hydroxyacyl-ACP, which is subsequently dehydrated to yield 2-enoyl-acyl-ACP. Finally, the C2–C3 double bond is reduced, yielding the elongated acyl chain (C_*n*__+__2_). After several reaction cycles, the end product acyl chain is transferred from ACP to acyl-CoA.

Cells require a plethora of acyl chains of varying length and unsaturation to establish different membrane lipid molecular species, which play a key role in maintaining membrane physical properties (Harayama and Riezman, 2018; Renne and de Kroon, 2018). FA synthesis only produces saturated FAs, mainly C16:0 or C18:0; therefore, cells harbor specific enzymes that can be elongated and/or desaturate acyl-CoA (Maier et al., 2006). The acyl-CoA chain length can be increased by elongase enzymes. FA elongation requires multiple enzymes and follows a multi-step process similar to FA synthesis by FAS. In contrast to FAS, enzymes of FA elongation are ER-localized.

In addition to synthesizing FAs *de novo*, cells can utilize dietary lipids or their own storage lipids. The lipids are hydrolyzed to provide FAs, which are subsequently activated by ligation to coenzyme-A. Synthesis of acyl-CoA is catalyzed by acyl-CoA synthetases (ACS/ACSL in mammalian cells, FAA in budding yeast). There are 13 different ACS isoforms in mammals that function in different tissues and subcellular locations (Grevengoed et al., 2014). The movement of the acyl-CoAs within cells is thought to be highly compartmentalized, yet how this occurs remains obscure (Cooper et al., 2015). ACSLs are found on the plasma membrane, ER, mitochondria and peroxisomes. In yeast, the main FAAs are soluble proteins, but are also found on the ER and on lipid droplets depending on yeast growth and nutritional state (Hariri et al., 2018).

### Lipid Biosynthesis and Turnover

The bulk of cellular FAs are incorporated into membrane lipids and neutral lipids (also known as storage lipids). Acyl-CoA and FFAs are only minor components of the cellular lipidome. Therefore, lipid biosynthesis is a major player in FA metabolism. FAs are incorporated in two major membrane lipid families: glycerophospholipids and sphingolipids.

Glycerophospholipids (GPL) are bulk constituents of membranes, thus a major sink for acyl chains. Most steps in GPL biosynthesis take place in the ER. The precursor to all GPLs is phosphatidic acid (PA), which is produced by acylation of glycerol-3-phosphate (G3P) at the *sn*-1 and *sn*-2 positions subsequently, catalyzed by ER localized acyltransferases (GPAT and AGPAT enzymes, respectively). PA can be either activated to CDP-diacylglycerol by CDP-DAG synthase or dephosphorylated to diacylglycerol (DAG) by PA hydrolase (Pah1 in yeast, Lipin in mammals) (Smith et al., 1957; Han et al., 2006). CDP-DAG is utilized to produce bulk GPLs such as phosphatidylcholine (PC), phosphatidylethanolamine (PE), and phosphatidylinositol (PI). In addition, PC and PE can be produced from DAG via the Kennedy-pathway (Kennedy and Weiss, 1956). Alternative to PL biosynthesis, DAG can be esterified to yield the neutral lipid TAG (as described below). Lipin/Pah1 serve as master regulators of lipid flux toward synthesis of membrane lipids (via CDP-DAG) or neutral lipids (via DAG), and their activity is tightly regulated by phosphorylation (O’Hara et al., 2006; Harris et al., 2007; Peterson et al., 2011). For example, yeast Pah1 is a cytosolic protein in its phosphorylated state, and dephosphorylation by the Nem1/Spo7 phosphatase complex is required for ER association and enzymatic activity (Siniossoglou et al., 1998).

Sphingolipid synthesis starts with the formation of the sphingosine backbone. First, 3-ketodihydrosphingosine is formed by condensation of serine and palmityl-CoA, catalyzed by serine palmitoyltransferase (SPT). The keto-group of 3-ketosphinganine is reduced to an alcohol, yielding sphinganine, which is *N*-acylated to yield the basic sphingolipid ceramide. In metazoans, ceramide is the precursor to simple sphingolipids, such as sphingomyelin, and complex glycosphingolipids such as cerebrosides and gangliosides. In yeast, ceramide is the precursor to inositol-phosphoceramide and derived glycosphingolipids.

In addition to membrane lipids, FAs are incorporated in storage lipids. This process takes place mainly in the ER. Storage lipids are synthesized by *O*-acylation of their respective precursors. In yeast and mammals these are mainly TAG and SE, synthesized from DAG and sterols by DGAT and SAT enzymes, respectively. In addition, there are minor storage lipids such as *O*-acyl ceramides (Voynova et al., 2012; Senkal et al., 2017) and retinol esters (Blaner et al., 2009; Orban et al., 2011; Molenaar et al., 2021).

### Fatty Acid Catabolism: β-Oxidation

To be used for energy production fatty acids must be broken down to acetyl-CoA, which can enter the citric acid cycle. This process is known as β-oxidation, named after oxidation of the acyl-CoA β-carbon. In yeast, β-oxidation takes place in peroxisomes, whereas in higher eukaryotes both peroxisomes and mitochondria are required. In metazoans, medium- and long chain FAs (C_*n*_ < 22) are oxidized in mitochondria, yielding acetyl-CoA. Very long chain FAs (C_*n*_ > 22) and branched chain FAs cannot be handled by mitochondria, and are first partially oxidized in peroxisomes, after which intermediate short chain FAs are transferred to mitochondria for the final oxidation steps.

FA β-oxidation follows the same enzymatic steps in mitochondria and in peroxisomes. First, FAs are activated to acyl-CoA, after which they enter a cycle of dehydrogenation, hydration, oxidation, and finally thiolysis. Each cycle shortens the acyl-CoA by 2 carbon atoms, yielding one acetyl-CoA. After oxidation of the FA β-carbon, the acyl chain remains only 2 carbon atoms long, providing another acetyl-CoA as end-product. Odd chain FAs yield propanoyl-CoA as end-product, which must be further processed to succinyl-CoA to enter the citric acid cycle.

## FA Metabolic Hubs at LD-Organelle Contact Sites

### LDs and the ER: Site of LD Biogenesis and Metabolic Channeling of FAs for Storage

It is well established that LDs originate from the ER, but the exact mechanisms of LD biogenesis remain to be fully elucidated. However, over the last years consensus has been reached on a model of LD biogenesis (Walther et al., 2017; Olzmann and Carvalho, 2019; Renne et al., 2020). In the prevalent model, neutral lipids produced by ER-resident enzymes coalesce between the phospholipid leaflets, forming “lens-like” structures (Thiam and Ikonen, 2021). Upon accumulation of neutral lipids, these lenses grow to form nascent LDs, which directionally bud toward the cytoplasm.

Formation of LDs is dependent on the synthesis of neutral lipids (Sandager et al., 2002). LDs are thought to originate from specialized ER subdomains, enriched in proteins required for proper LD formation (Joshi et al., 2018; Wang et al., 2018; Salo et al., 2019; Choudhary et al., 2020). Yeast Pah1 was found to be enriched at LD biogenesis sites at the ER and the NVJ, suggesting that local DAG synthesis is involved in LD formation (Adeyo et al., 2011; Barbosa et al., 2015b). In addition, the TAG biosynthetic enzyme Lro1p was shown to localize to ER-sites of LD formation upon induction of neutral lipid (NL) biosynthesis (Choudhary et al., 2020). In mammalian cells, ACSL3 has been shown to be recruited to LD biogenesis sites, likely to provide local synthesis of acyl-CoA substrate required for NL formation (Kassan et al., 2013). Recruitment of ACSL3 to LD biogenesis sites is severely impaired by loss of Seipin, indicating a role for Seipin in establishing the sites of LD formation (Salo et al., 2016). In agreement with this model, the formation of discrete LD biogenesis domains in the ER in yeast was shown to depend on Seipin and the Pah1-phosphatase Nem1 (Choudhary et al., 2020).

How neutral lipids produced in the ER are packaged into LDs, is a key question to be solved. The ER protein Seipin has been implicated in facilitating this process. Seipin plays a key role in LD biogenesis (Szymanski et al., 2007; Fei et al., 2008), but it’s function has been elusive. Great advances were made recently by several studies that employed structural, *in silico*, biochemical, biophysical, and cell biological approaches. High resolution structures showed that human and Drosophila Seipin adopt a ring-like structure, mediated by protomer interactions in the ER lumenal domain (Sui et al., 2018; Yan et al., 2018). The ring structure formed by the protomer β-sandwiches is oriented parallel to the membrane, whereas the α-helices point toward the membrane. Recent *in silico* data indicates that these α-helices protrude into the membrane and bind TAG, likely to facilitate efficient packaging into LDs (Zoni et al., 2019; Prasanna et al., 2021).

ER-LD contacts are likely key sites of protein and lipid exchange between the ER and LDs and are important for LD maintenance and response to cellular metabolic state. During LD expansion the main determinant for LD size is the amount of neutral lipids produced, and several lipid metabolic enzymes have been identified on the LD surface, to facilitate local NL synthesis (Wilfling et al., 2013; Currie et al., 2014; Bersuker et al., 2018). Under such conditions, the acyltransferases GPAT3, AGPAT3, and DGAT2 are recruited from the ER to LDs (Wilfling et al., 2013), and this re-localization likely takes place at ER-LD contacts. Indeed, in *C. elegans* the LD-localized DGAT2 activity seems to be coordinated with the activity of the ER-localized acyl-CoA synthethase FATP1, and these contacts are required for LD expansion (Xu et al., 2012). Interestingly, LDs can form and grow in the absence of LD-localized NL synthesis. Mammalian DGAT1 and yeast LCAT (Lro1p) are ER-localized proteins, both having their catalytic site in the ER lumen (McFie et al., 2010; Choudhary et al., 2011). Expression of Lro1p as sole neutral lipid biosynthetic enzyme is sufficient for formation of LDs without apparent defects in size or number (Sandager et al., 2002). Thus, NLs formed in the ER must be able to be efficiently targeted to LDs, and this process is likely facilitated by ER-LD contact sites.

The ER and LDs form stable contact sites, and both organelles have been observed to be connected via lipidic bridges LDs (Jacquier et al., 2011). Stability of these ER-LD contacts depends on Seipin in yeast and mammals (Grippa et al., 2015; Salo et al., 2016), indicating a role for Seipin in maintaining ER-LD contact sites. Furthermore, Seipin has been shown to interact with various enzymes involved in NL formation, including glycerol-3-phosphate acyltransferase (GPAT)-, acylglycerol acyltransferase (AGPAT)-, and PA hydrolase (PAH) enzymes (Sim et al., 2013; Pagac et al., 2016). This observation provides the possibility that these enzymes are localized to ER-LD contact sites, providing local DAG synthesis at ER-LD contacts (Figure 1). Interestingly, Seipin was recently shown to inhibit the synthesis of serine palmitoyltransferase (SPT), which mediates the first step in the synthesis of sphingolipids, at ER-LD contact sites (Su et al., 2019). As sphingolipids are not used for the synthesis of bulk neutral lipids, it is unlikely that this inhibition is related to regulation of neutral lipid biosynthesis. This observation could point to a role for Seipin as a general regulator of lipid metabolism at ER-LD contact sites.

LDs are thought to form at defined locations on the ER membrane. What determines the sites of formation of nascent LDs on the ER membrane is not completely understood. Recent work revealed that MCTP1 and 2 (multiple C2 domain-containing transmembrane proteins) colocalize with seipin and promote LD biogenesis at specialized microdomains within ER tubules. These proteins mediate stable ER-LD contact sites thus regulating LD biogenesis, number, and size (Joshi et al., 2021). Several other proteins have been recently proposed to function as tethers between the ER and LDs in addition to Seipin. In mammalian cells where LD expansion is stimulated by exposing cells to exogenous FAs, the ER-localized Snx14 is be recruited to ER-LD contacts in a Seipin-independent fashion (Datta et al., 2020). Snx14 accumulates at ER sites that are enriched in the FAA enzyme ACSL3 and promotes TAG synthesis in a ASCL3 dependent manner. Whether Snx14-stimulated TAG synthesis requires specific GPAT, AGPAT, and DGAT enzymes is not known. The Rab GTPase Rab18 establishes LD contacts with the ER (Ozeki et al., 2005), and these contacts depend on the NAG-RINT1-ZW10 (NRZ) tethering-complex and associated SNARE proteins and the Rab18 binding partner DFCP1 (Xu et al., 2018; Li et al., 2019). Overexpression of DFCP1 increases ER-LD contacts, and when Rab18 is overexpressed LDs are strikingly wrapped by the ER (Li et al., 2019). In adipocytes, loss of Rab18 leads to aberrant LD formation and a dramatic increase of LD size (Xu et al., 2018), further underscoring the importance of ER-LD contact sites in LD maintenance. Interestingly, the lipid transport proteins VPS13A and VPC13C have also been found to localize to ER contacts with mitochondria, LDs or lysosomes (Kumar et al., 2018; Yeshaw et al., 2019), and likely play a role in exchanging bulk lipids at these contacts during organelle expansion.

The endoplasmic reticulum is spread throughout the cell, with tubules stretching from the center of the cell toward the plasma membrane, and it forms contacts with all cellular organelles (Wu et al., 2017). A twist to contacts between the ER-LD contacts recently came with the that LDs could localize at contacts between the ER and other organelles, creating an organelle trijunction. In yeast, LDs were found to localize to the nuclear vacuole junction, a contact site between the nuclear ER and the vacuole (discussed in detail below). In the fly fat body, LDs are also found throughout the cell and two distinct LD populations can be identified; one localizing more toward the center of the cell, the other more toward the cell periphery. Interestingly, peripheral LDs were recently shown to spatially organized by Snazarus (Snz), that connects LDs to ER-PM contact sites (Ugrankar et al., 2019). The peripheral LDs were observed to be smaller than the central LDs and were found to be metabolically distinct. These observations underscore the notion that metabolically distinct subpopulations of LDs may exist within the same cell and implicate LD contact sites in regulation of these subpopulations.

### LDs and Peroxisomes/Mitochondria: Coordination of FA Flux Toward β-Oxidation

To facilitate β-oxidation, FAs have to be released from the NLs stored in LDs and delivered to either peroxisomes or mitochondria (Figure 1). NL catabolism can occur via lipolysis, catalyzed by cytosolic lipases, or through lipophagy, in which LDs are degraded in lysosomes (mammals) or the vacuole (yeast). The yielded FFA are then transferred to the mitochondria or peroxisomes. How FA are released from the LD core and directed toward mitochondria and peroxisomes remained enigmatic. Recent advances indicate that FA transport from LDs to peroxisomes/mitochondria may be mediated by LD-mitochondrion and LD-peroxisome contact sites. Direct handover of released FAs is thought to be an efficient way to prevent accumulation of excess fatty acids, thereby omitting possible lipotoxicity.

In yeast and plants, peroxisomes are the sole organelles that perform β-oxidation, whereas in metazoans they are responsible for β-oxidation of very long-chain FAs. Therefore, LDs and peroxisomes must collaborate to regulate flux and availability of FAs within the cell (Kohlwein et al., 2013). Close physical associations between LDs and peroxisomes have been observed in mammals, yeast, and plants (Schrader, 2001; Binns et al., 2006; Gao and Goodman, 2015). When yeast cells are grown on oleate as sole carbon source, peroxisome numbers are increased and they form persistent LD-pex contact sites, indicating tight collaboration between these organelles. Interestingly, peroxisomes have been observed to form extensions that can protrude into LDs (Binns et al., 2006). As LD-peroxisome contact sites are thought to play a role in facilitating local FA trafficking among these organelles, these pexopodia could serve as a manner to increase the contact surface between these two organelles.

The topological details and molecular machinery that tether LDs and peroxisomes has remained largely unknown. Recent work demonstrated that the AAA-ATPase M1 Spastin found on LDs can directly interact with the peroxisomal ATP binding cassette subfamily D member 1, ABCD1. This interaction regulates LD-peroxisome contacts and promotes FA inter-organelle exchange (Chang et al., 2019). Interestingly, this study showed that M1 Spastin recruit the membrane-shaping ESCRT-III proteins, which are thought to modify LD membrane morphology to facilitate lipid movement (Chang et al., 2019). LD-peroxisome contacts were found to be important to maintain energy homeostasis during fasting. Specifically, peroxisomal protein PEX5 was found to escort adipose triglyceride lipase ATGL to LDs to mediate fasting-induced lipolysis (Kong et al., 2020). LD-peroxisome tethering also forms by the interaction of peroxisomal acyl-CoA binding domain containing 5 (ACBD5) and ER-localized VAPs (VAP-A and VAP-B) (Costello et al., 2017; Hua et al., 2017). Disruption of this tethering complex was found to alter peroxisome-ER contacts and prevent the growth of peroxisomal membrane suggesting that this contact site is required to transfer lipid from the ER to peroxisomes (Schuldiner and Zalckvar, 2017; Chen et al., 2020).

Understanding of the physiological roles of LD-peroxisome contact sites in localized FA trafficking and metabolism requires more intense investigation. Specifically, understanding how disruption of LD-peroxisome contact sites contributes to human peroxisomal disorders is still unclear. Peroxisomal function is also known to be altered in aging, thus contributing to a host of age-related diseases including diabetes, neurodegeneration, and cancer (Titorenko and Terlecky, 2011; Lodhi and Semenkovich, 2014; Cipolla and Lodhi, 2017).

In metazoans, mitochondria are a key site for β-oxidation. FA β-oxidation in mitochondria requires release of FAs from the LD through lipolysis and transfer to mitochondria, where they are imported via carnitine shuttling. As such, LD-mitochondria contact sites are highly apparent in cells with high fatty acid oxidation rates, including brown adipose tissue and muscle cells. In skeletal muscle cells, LDs have been observed to be “sandwiched” between mitochondria, likely to increase the contact surface, and these drastic LD-mitochondria contacts have been proposed facilitate the high demand of FA trafficking for rapid energy production (Shaw et al., 2008).

In agreement with LD-contacts facilitating FA handover to mitochondria, LD-mitochondria contacts have been observed to respond to metabolic state and the need for FA oxidation. For example, when cells are acutely starved, organelles are broken down via autophagy, releasing free fatty acids as lipid breakdown products. The released FAs are efficiently incorporated into LDs, and are handed over from LDs to mitochondria for β-oxidation (Nguyen et al., 2017). Under starvation conditions, LDs were often found in close proximity to mitochondria, likely to facility efficient FA trafficking (Nguyen et al., 2017). Indeed, close apposition of mitochondria to LDs has previously been proposed to be required for efficient flux of FAs from LDs into mitochondria (Rambold et al., 2015). Thus, LD-mitochondria contact sites respond to cellular metabolic state to coordinate FA mobilization, handover, and oxidation. Supporting this model, LD-mitochondria contact sites are highly apparent in cells with high FA oxidation rates, such as brown adipose tissue and muscle cells.

Interestingly, contacts with LDs may dictate mitochondrial metabolism, as mitochondria that are in contact with LDs have been shown to form a subpopulation that is biochemically distinct from cytosolic mitochondria. The proteome of LD-approximate mitochondria was found to differ from cytosolic mitochondria, and both subpopulations partake in different metabolic processes (Benador et al., 2018). Strikingly, in this study it was found that LDs that are in contact with mitochondria increase TAG synthesis for LD expansion, and that cytosolic mitochondria mainly partake in beta oxidation (Benador et al., 2018, 2019), which is at odds with the model that LD-mitochondria contacts are required for mitochondrial FA oxidation. These differences could be explained by cell type specificity, as Rambold et al. (2015) use cultured mouse embryonic fibroblasts whereas Benador et al. (2018) used primary mouse brown adipose tissue, or by the different methods used to stimulate FA oxidation. Additionally, the differences in metabolic state of cultured cells vs. primary derived tissue is likely to influence LD and mitochondrial dynamics and metabolism, complicating the comparison between these studies. The interplay between mitochondria and LDs is an intriguing topic, and the role of these contacts and how they are regulated remains to be fully elucidated.

Several proteins have been reported to play a role in LD-mitochondria contact site formation (Schuldiner and Bohnert, 2017), but the mechanisms facilitating local FA channeling remain to be fully elucidated. For example, the perilipin family member PLIN5 overexpression is sufficient to induce an increase in LD-mitochondria contact sites (Wang et al., 2011; Benador et al., 2019). In brown adipocytes, PLIN1 interacts with the outer mitochondrial membrane protein mitofusin 2 (MFN2) potentially forming a tethering complex which is stimulated under lipolytic conditions (Boutant et al., 2017). More recently, a new ESCRT-dependent mechanism by which FAs are trafficked from LDs to mitochondria was described. This was shown to be mediated by the lipid transfer protein VPS13D and the ESCRT protein Tsg101 (Wang et al., 2021). Finally, the observation of LD-mitochondria contact sites in yeast, where β-oxidation takes place solely in peroxisomes (Pu et al., 2011), as well the notion that several lipid metabolic enzymes can localize to both mitochondria and LDs in yeast), indicate that LD-mitochondria contacts likely have lipid metabolic functions beyond channeling of FA for β-oxidation.

### Localized FA Storage and Mobilization at LD-Lysosome Contacts

Lysosomes play a central role in the recycling of organelles and biomolecules, and contain hydrolytic enzymes that can breakdown proteins, nucleic acids, and lipids. In yeast, LDs can be consumed by micro-autophagy (or micro-lipophagy), during which LDs are directly internalized in the vacuole (the yeast lysosome) and degraded by the internal hydrolases (Toulmay and Prinz, 2013; Wang et al., 2014). This process occurs in response to various starvation conditions including nitrogen starvation and acute glucose starvation (Seo et al., 2017). It remains to be elucidated whether a similar mechanism of micro-lipophagy is responsible for LD internalization and lysosomal degradation in mammals. Recent work in hepatocytes revealed that interactions between mammalian lysosomes and LDs facilitate the direct transfer of proteins and lipids to lysosomes (Schulze et al., 2020). This suggest that the mammalian lysosomes are indeed sufficient to mediate LD turnover independent of an autophagosomal intermediate (Drizyte-Miller et al., 2020; Schulze et al., 2020). Additionally, interactions between LDs and lysosomes or autophagosomes are important not only for LD degradation, and they also supply membrane lipids for autophagosome biogenesis (Schütter et al., 2020).

In yeast, interactions between LDs and vacuoles (lysosome equivalent in mammalian cells) have been shown to be tightly regulated by metabolic state. During diauxic shift, when glucose is exhausted and yeast cells switch from fermentative growth to aerobic restoration, the size of the vacuole is increased. During this stage, they also form a unique contact site between the nucleus and the vacuole, dubbed the nuclear-vacuole junction (NVJ), and LDs start to localize there (Barbosa et al., 2015b; Hariri et al., 2018; Figure 1). The NVJ is proposed to function as a metabolic platform that spatially organizes acyl chain metabolism in response to nutrient depletion. During the diauxic shift, when, NVJ contact sites expand in size by upregulating protein levels of Nvj1, a major tether at the NVJ. Concomitantly, LDs are observed to enrich at the NVJ. How sub-populations of LD form locally at the NVJ is an ongoing topic of investigation.

Enrichment of enzymes responsible for neutral lipid biosynthesis at the NVJ is likely to drive local LD biogenesis. Indeed, during diauxic shift the fatty acid-CoA ligase Faa1 is targeted to the NVJ. By locally activating free fatty acids, yielding acyl CoA, Faa1 likely provided acyl-CoA substrate required for the formation of neutral lipids. In addition, other lipid metabolic enzymes (such as Tsc13 and Pah1) have also been described to be recruited to the NVJ in different conditions (Kohlwein et al., 2001; Barbosa et al., 2015a). These observations partake only single enzymes from lipid metabolic pathways, but they strongly suggest that the NVJ contributes to the spatial compartmentalization of TG biosynthesis metabolons, though this remains to be demonstrated (Henne et al., 2020).

How NVJ targeting is controlled remains an open question. Recently, Faa1 was found to interact with the NVJ tether Mdm1, likely providing the mechanism of Faa1 targeting to the NVJ and subsequent local LD formation (Hariri et al., 2018). Supporting this, over-expression of Mdm1 drives the accumulation of LDs at the NVJ. Interestingly, the PXA (Phox homology-associated) domain of Mdm1 binds FAs *in vitro*. Therefore, Mdm1 provides a scaffolding function that facilitates the generation of a high local concentration of activated FAs and promotes their incorporation into neutral lipids (Hariri et al., 2019).

Mdm1 is conserved in fruit flies (Drosophila melanogaster) as Snazarus (Snz), which is highly expressed in the Drosophila fat body (FB) and was originally associate with a longevity phenotype (Suh et al., 2008). Similar to Mdm1, Snz is an ER-anchored protein that binds to LDs. However, this study revealed that Snz localizes to ER-PM contacts and maintains a specific subpopulation of LDs near the cell periphery (Ugrankar et al., 2019). As such, Snz is thought to coordinate FA uptake with TG synthesis and LD growth. Mdm1 is conserved in humans as four sorting nexins (Snx) 13, 14, 19, and 25 (Henne et al., 2015). Less is known about the human homologs of Mdm1/Snz. Snx14 was shown to localize to ER-LD contact sites and facilitates FA-to-TG conversion, and like Mdm1, loss of Snx14 sensitizes cells to FA-induced lipotoxicity (Datta et al., 2019). Loss of Snx13 has been implicated in heart failure; however, whether altered FA metabolism contribute to the pathology is currently an open question (Li et al., 2014; Yang et al., 2019).

## Open Questions and Future Directions

LDs are reservoirs of FAs that are found in organisms from microbes to humans. Survival of organisms in nutrient scarce conditions depends on their ability to mobilize and utilize their stored FAs. FAs have multiple cellular fates and following their mobilization they are targeted to different cellular compartments to be processed. How FAs are trafficked among organelles remains a key question in biology. LDs form inter-organelle contacts that are thought to facilitate efficient trafficking of FAs. At the core of these contacts are tethering proteins that physically hold these organelles together, in addition to lipid metabolic enzymes and transfer proteins that meditate inter-organelle lipid movement. Arguably, the least understood contact site is the one that forms between LDs and peroxisomes. Emerging technologies such as enzyme-mediated proximity labeling will be useful to determine the full proteome of LD contact sites.

The size, number and cellular distribution of LDs is highly dynamic, and can vary according to metabolic cues. However, the regulatory mechanisms controlling LD formation, mobilization and turnover remain largely unknown, including how LD recruitment to specific contact sites is orchestrated. As LDs play a central role in lipid homeostasis, it is attractive to speculate a role for lipid signaling in dictating LD dynamics. Various proteins found at LD contact sites and (proposed) tethers possess (putative) lipid binding domains, including the phosphoinositide binding Pleckstrin homology domain found on VPS13A/C, and Phox homology domains on Mdm1 and Snazarus (Hariri et al., 2018; Kumar et al., 2018; Ugrankar et al., 2019). Peroxisomal PI-4,5-P_2_ has been shown to be required for trafficking of very long chain FAs from LDs to peroxisomes (Ravi et al., 2021), possibly by regulating LD-Peroxisome contact sites or recruiting lipid metabolic enzymes. Recently, the lipid transfer protein ORP5 was shown to localize to ER-LD contact sites and regulate LD PI-4-P levels (Du et al., 2019), giving rise to the possibility of phosphoinositide signaling on LDs (Renne and Emerling, 2020).

Future research will rely on the development of reliable tools to monitor FA trafficking in cells. Beyond their role in facilitating direct FA transfer, LD contact sites are viewed as dynamic platforms that play central roles in regulating FA metabolism. *In vitro* reconstitution of FA metabolism at organelle contacts will allow for better understanding of how spatial organization of enzymes fine tune the efficiency and outcome metabolic reactions. Finally, the contribution of LD contact sites to cellular lipid homeostasis and organismal health is currently not completely understood. A key question that remains to be answered is how the disruption of inter-organelle networks leads to metabolic disorders of FA metabolism.

## Author Contributions

HH and MR contributed to writing and discussing ideas. MR designed the figure. Both authors contributed to the article and approved the submitted version.

## Conflict of Interest

The authors declare that the research was conducted in the absence of any commercial or financial relationships that could be construed as a potential conflict of interest.

## Publisher’s Note

All claims expressed in this article are solely those of the authors and do not necessarily represent those of their affiliated organizations, or those of the publisher, the editors and the reviewers. Any product that may be evaluated in this article, or claim that may be made by its manufacturer, is not guaranteed or endorsed by the publisher.
